# Shared decision making with oncologists and palliative care specialists effectively increases the documentation of the preferences for do not resuscitate and artificial nutrition and hydration in patients with advanced cancer: a model testing study

**DOI:** 10.1186/s12904-020-0521-7

**Published:** 2020-02-04

**Authors:** Hsien-Liang Huang, Jaw-Shiun Tsai, Chien-An Yao, Shao-Yi Cheng, Wen-Yu Hu, Tai-Yuan Chiu

**Affiliations:** 1 Department of Family Medicine, College of Medicine and Hospital, National 7 Chung-Shan South Road, Taipei, 100 Taiwan; 2 School of Nursing, College of Medicine and Hospital, National Taiwan University, Taipei, Taiwan

**Keywords:** Shared decision making, Advanced care planning, Advanced cancer, Do not resuscitate, Artificial nutrition and hydration

## Abstract

**Background:**

Communication in do not resuscitate (DNR) and artificial nutrition and hydration (ANH) at the end of life is a key component of advance care planning (ACP) which is essential for patients with advanced cancer to have cares concordant with their wishes. The SOP model (Shared decision making with Oncologists and Palliative care specialists) aimed to increase the rate of documentation on the preferences for DNR and ANH in patients with advanced cancer.

**Methods:**

The SOP model was implemented in a national cancer treatment center in Taiwan from September 2016 to August 2018 for patients with advanced cancer visiting the oncology outpatient clinic. The framework was based on the model of shared decision making as “choice talk” initiated by oncologists with “option talk” and “decision talk” conducted by palliative care specialists.

**Results:**

Among 375 eligible patients, 255 patients (68%) participated in the model testing with the mean age of 68.5 ± 14.7 years (mean ± SD). Comparing to 52.3% of DNR documentation among patients with advanced cancer who died in our hospital, the rate increased to 80.9% (206/255) after the decision talk in our model. Only 6.67% (*n* = 17) of the participants documented their preferences on ANH after the model. A worse Eastern Cooperative Oncology Group Performance Status was the only statistically significant associating factor with a higher rate of DNR documentation in the multiple logistic regression model.

**Conclusions:**

The SOP model significantly increased the rate of DNR documentation in patients with advanced cancer in this pilot study. Dissemination of the model could help the patients to receive care that is concordant with their wishes and be useful for the countries having laws on ACP.

## Background

Advance care planning (ACP) is a process of open discussion on topics including the assignment of a medical durable power of attorney, living wills, personal values and preferences for end-of-life (EOL) care which is essential for patients to honor their autonomy in future medical decisions [1]. Communication on the preferences for life-sustaining treatments and artificial nutrition and hydration at EOL is a key component in ACP to ensure the quality of care in accordance with the patient’s wishes and to preserve the dignity at the EOL [2–4]. ACP is widely recognized by many professional bodies, and current guidelines regarding care of patients with advanced cancer, such as “Dying in America: Improving Quality and Honoring Individual Preferences Near the End of Life” from the Institute of Medicine [5] and “Integration of Palliative Care Into Standard Oncology Care” from the American Society of Clinical Oncology [6, 7], all recommend ACP as a required component for better care quality. For patients with advanced cancer, communication to help them understand the prognosis, symptom control, and treatment preferences is undoubtedly of high priority [8, 9]. The importance of documenting preference on EOL during ACP in these patients is demonstrated [10, 11], and the lack of advance directives regarding care options during EOL could lead to negative consequences for patients and caregivers [12–14]. Communication and documentation of EOL care preferences in ACP among cancer patients is also encouraged [4, 15]. Furthermore, Taiwan is the first in Asia to implement the Patient Right to Autonomy Act [16]. The Act, which is enacted in 2019, states that patients have the right to receive or refuse life-sustaining treatments and ANH in specific clinical conditions after ACP. Through the process of ACP, the Patient Right to Autonomy Act provides the legal basis to help the patients make decisions regarding the treatment options provided by the physician. However, previous surveys on decision making regarding EOL management with patient education tools such as video decision aids reported only about a 30–40% documentation rate of treatment preferences [11, 17–19]. Several attempts from oncologists including using electronic prompts to remind oncologist for ACP, creating new workflows to incorporate ACP into routines in oncology outpatient clinics, or using semi-structured discussion are implemented to increase the documentation of code status and the rate have been elevated to about 60–70% [20–22].

Shared decision making (SDM) as a way of engaging the patients and their families in the process of choosing a treatment strategy is increasingly endorsed to be the ideal model in the era of person-centered care [23, 24]. Basic components of SDM include informing the patients of reasonable options, providing detailed explanations with professional suggestions, exploring the patients’ preferences, and facilitating communication to support the patients in deliberation [25, 26]. Furthermore, with growing interests from policymakers and the public, physicians are urged to continuing incorporating SDM into clinical practice [27]. In order to improve patient-centered communication in ACP and the documentation of EOL preferences, incorporation of SDM into the ACP process might be an effective strategy.

On the other hand, early integration of palliative care into standard oncology care had been suggested for patients with advanced cancer by guidelines [6]. The integration of palliative care team and oncologists has been shown to benefit the patients in providing better patient-reported outcomes, EOL cares that are concordant with patients’ preferences, relief of psycho-social-spiritual distress, and mood support for patients and families [7, 28, 29]. Moreover, increased understanding of disease prognosis and assistance with decision making are the essential components of palliative care provided by interdisciplinary palliative care teams [6]. One study revealed that patients with advanced cancer involved in early palliative care are more likely to have communications to facilitate decision making in EOL care [29].

Previous studies have demonstrated that ACP could help patients with advanced cancer through the process of EOL treatment decisions [19, 30, 31]. Also, the communication regarding preferences for DNR and ANH at EOL as a part of ACP could assist cancer patients to receive medical care that is concordant with their wills. Hence, the documentation of DNR and ANH through the SDM process may be viewed as the initiation of earlier palliative care integration as the congruence of patients, family, and medical professionals on the choices of life-sustaining treatments at the EOL. With this backdrop, we established the SOP model (Shared decision making with Oncologists and Palliative care specialists) designed to translate the merits of SDM into clinical practice. The model was initiated by oncologists and followed up by communication with palliative care specialists. It aimed to increase the documentation rates of DNR and ANH preferences in patients with advanced cancer. The results may provide an effective approach to improve the processes of medical decision-making among patients with advanced cancer.

## Methods

### Framework and development of the SOP model (shared decision making with oncologists and palliative care specialists)

**Fig. 1:**
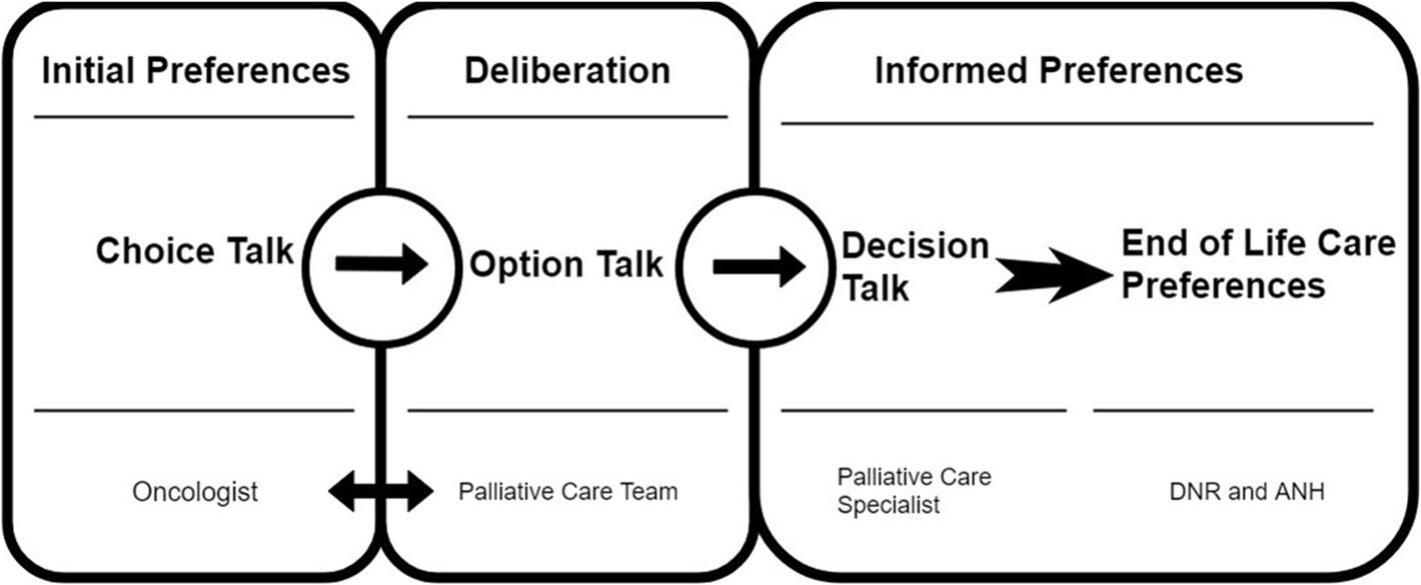
The framework of the SOP model (Shared decision making with Oncologists and Palliative care specialists)

The framework of the model was based on the traditional three talk model of SDM including “choice talk,” “option talk,” and “decision talk” with the integration of the concept of the SHARE model (Seek participation, Help comparison, Assess values, Reach decision, Evaluate decision) proposed by the Agency for Healthcare Research and Quality [25, 32, 33]. The step “choice talk,” including patient participation, providing treatment choices, and initial preference exploration, was initiated by the oncologists. The “option talk” focused on the information exchange specifically in EOL treatment preferences regarding DNR and ANH between the palliative care team and patients and their families. The “decision talk” was designed to reach decisions on the treatment preferences on DNR and ANH in EOL condition after assessing the patients’ values (Fig. 1).

The goal of the “choice talk” was for the physicians to make clear to the patients all the different treatment choices for the condition, and the involvement of patients’ opinions are also essential for further SDM. For patients with advanced cancer, treatment discussions must include the provision of palliative care and communication on EOL care preferences [30]. At the same time, detailed exploration and understanding of EOL treatment options such as ACP were too time-consuming for oncologists especially considering there were other treatment decisions to be made. Therefore, oncologists only provided palliative care consultation with further ACP as one of the treatment choices in the model.

The “option talk” was designed to aid the decision with descriptions of harms and benefits for the treatment choices [33]. In the SOP model, this step consisted of a pamphlet and discussion with a palliative care specialist. The decision aid in the form of a pamphlet was given to patients to read for 10 min which was followed by a semi-structured discussion for 20 min. The pamphlet served as a decision support tool which was structured in three parts consisting of an overview of the pamphlet with descriptions on the importance of ACP, the pros and cons of EOL care options (especially on DNR and ANH), and questions that help the patients to clarify their preferences toward the treatment choices. The pamphlet was developed according to GRADE (Grading of Recommendations Assessment, Development and Evaluation) and the requirement for patient decision aids in ethics, quality-of-care, and evidence-based medicine [34, 35]. During the development phase, it was reviewed by five oncologists, five palliative care specialists, ten nurses, one social worker, and five administrative staff. Also, five volunteers and five patients with advanced cancer tested the face validity of the pamphlet. The discussion was semi-structured (see interview guide in Additional file 1) with three themes: (1) rapport building and communication between patients and physicians; (2) symptoms and adverse effects due to current anti-cancer treatment; (3) EOL care preferences. Questions about EOL care preferences included: “Have you considered the care preferences toward the EOL such as whether you would choose to be cardio-pulmonary resuscitated?” “Have you considered to receive clinically assisted hydration via nasogastric tube, gastrostomy, or intravenous administration toward the EOL?”. The semi-structured discussion was also pre-tested as described for the pamphlet.

The goal of the “decision talk” was to make decisions based on the patients’ will after deliberation [2, 3]. Essential components of palliative care included establishing rapport with the patients and exploring the patients’ understanding of the illnesses with treatment preferences [6]. Palliative care specialists elicited the patients’ preferences, and the preferences for DNR and ANH were documented in this final step of the model.

### Implementation of the SOP model

The SOP model was implemented in National Taiwan University Hospital, a national cancer treatment center in Taiwan. It was conducted in an outpatient clinic setting, and the current analysis was conducted from September 2016 to August 2018. Patients with solid tumors visiting the oncology outpatient clinic were eligible to enroll if they were more than 20 years old of age, were able to understand spoken Chinese, were clearly competent, were able to communicate on inform consent, had an estimated life expectancy of 3 to 12 months, and were recommended by oncologists that the integration of palliative care was needed for the management of refractory pain or other symptoms, management of more complex psycho-spiritual distress, and assistance with conflict resolution of treatment goals [1, 36].

Information on ACP was provided to patients during the communication of treatment choices in the outpatient oncology clinic. Patients were enrolled in the model if they were willing to join the next step of the “option talk” to receive further information on DNR and ANH. After the agreement on enrollment, visits to the palliative care clinic was arranged within two weeks for the “option talk” and “decision talk”. We also recommended patients to visit the palliative clinic accompanied by at least one family member or main caregiver. The “option talk” and “decision talk” took about 30 min and 10 min, respectively. The model focused on the patients’ EOL care preferences on DNR and ANH. The items of DNR included intubation, cardiac massage, inotropes using, defibrillation, and tracheostomy. The choices of ANH included nasogastric tube, gastrostomy, or intravenous fluids. In the “decision talk,” preferences on DNR and ANH were then documented with patients’ signatures even the patient chose to be resuscitated or receiving ANH. The DNR documentation form was then uploaded to the national electronic health record by social workers if the patient chose not to be resuscitated, and the ANH preferences were documented in the hospital electronic information system by palliative care specialists. The DNR documentation rate was compared for patients with advanced cancer who died in our hospital during the study period based on administrative data.

### Statistical analysis

Demographic characteristics were analyzed by conducting the independent t-tests for numeric data, while using Chi-Square tests and Fisher’s exact tests for categorical data. Mean ± Standard deviation and counts (percentage) were presented in Table 1. Both univariate and multivariable analyses were conducted by logistic regression to address the association between completing DNR and variables of interest. SAS 9.4 was used in this study, and a *p*-value of less than 0.05 was considered as statistically significant.

**Table 1:** Demographic characteristics of patients (*n* = 255)

	Overall (*n* = 255)	DNR documentation		
		Completed (*n* = 206)	Not completed (*n* = 49)	*p*
Age	68.49 ± 14.67	68.55 ± 14.80	68.24 ± 14.27	0.895
Gender				0.131
Male	134 (52.55%)	113 (84.30%)	21 (15.70%)	
Female	121 (47.45%)	93 (76.86%)	28 (23.14%)	
Education				0.030
Elementary school or below	86 (33.73%)	65 (31.55%)	21 (42.86%)	
Junior high school	30 (11.76%)	20 (9.71%)	10 (20.41%)	
Senior high school	65 (25.49%)	56 (27.18%)	9 (18.37%)	
University or above	74 (28.63%)	65 (31.55%)	9 (18.37%)	
Marital status				0.675
Married	185 (72.55%)	148 (71.84%)	37 (75.51%)	
Widowed	37 (14.51%)	29 (14.08%)	8 (16.33%)	
Single	21 (8.24%)	19 (9.22%)	2 (4.08%)	
Divorced/separated	12 (4.71%)	10 (4.85%)	2 (4.08%)	
Primary site of cancer				0.459
Urogenital	88 (34.51%)	66 (32.04%)	22 (44.90%)	
Gastrointestinal	47 (18.43%)	38 (18.45%)	9 (18.37%)	
Respiratory	30 (11.76%)	23 (11.17%)	7 (14.29%)	
Head and neck	29 (11.37%)	25 (12.14%)	4 (8.16%)	
Skin	8 (3.14%)	6 (2.91%)	2 (4.08%)	
Breast	6 (2.35%)	5 (2.43%)	1 (2.04%)	
Other or unknown	47 (18.43%)	42 (20.39%)	5 (10.20%)	
Metastasis				0.156
Without	57 (22.35%)	41 (19.90%)	16 (32.65%)	
With	149 (58.43%)	124 (60.19%)	25 (51.02%)	
Unknown or multiple sites	49 (19.22%)	41 (19.90%)	8 (16.33%)	
ECOG				< 0.001
0 and 1	95 (37.25%)	66 (32.04%)	29 (59.18%)	
2	91 (35.69%)	75 (36.41%)	16 (32.65%)	
3	69 (27.06%)	65 (31.55%)	4 (8.16%)	
Religion				0.153
Taoism/traditional religions	83 (32.55%)	65 (31.55%)	18 (36.73%)	
Buddhism	80 (31.37%)	66 (32.04%)	14 (28.57%)	
Christianity	23 (9.02%)	20 (9.71%)	3 (6.12%)	
Not specified	62 (24.31%)	51 (24.76%)	11 (22.45%)	
Other	7 (2.75%)	4 (1.94%)	3 (6.12%)	

Abbreviations: *DNR* do not resuscitate; *ECOG* Eastern Cooperative Oncology Group performance status

## Results

Between September 2016 and August 2018, 375 patients with advanced-stage solid tumors met the inclusion criteria and were asked to participate. A total of 255 patients (68%) were willing to participate in the model. The characteristics of the participants are presented in Table 1. Overall, the mean age was 68.5 ± 14.7 years (mean ± SD) and 121 patients (47.5%) were female. Different education levels were well represented among the participants. The primary cancer locations of urogenital (34.5%), gastrointestinal (18.4%), respiratory (11.8%), and head-and-neck (11.4%) accounted for more than 70% of the participants. There were 47 patients (18.4%) classified as “other or unknown” because these patients had other tumor origins or did not have tissue proof for definitive diagnoses. Among the participants, 37.3% had the ECOG (Eastern Cooperative Oncology Group) performance status 0 or 1, 35.7% was status 2, and 27.1% was status 3. As to religion, more than 60% of the participants believed in Buddhism, Taoism, and traditional religions while 23 patients were Christian (9.02%).

**Fig. 2:**
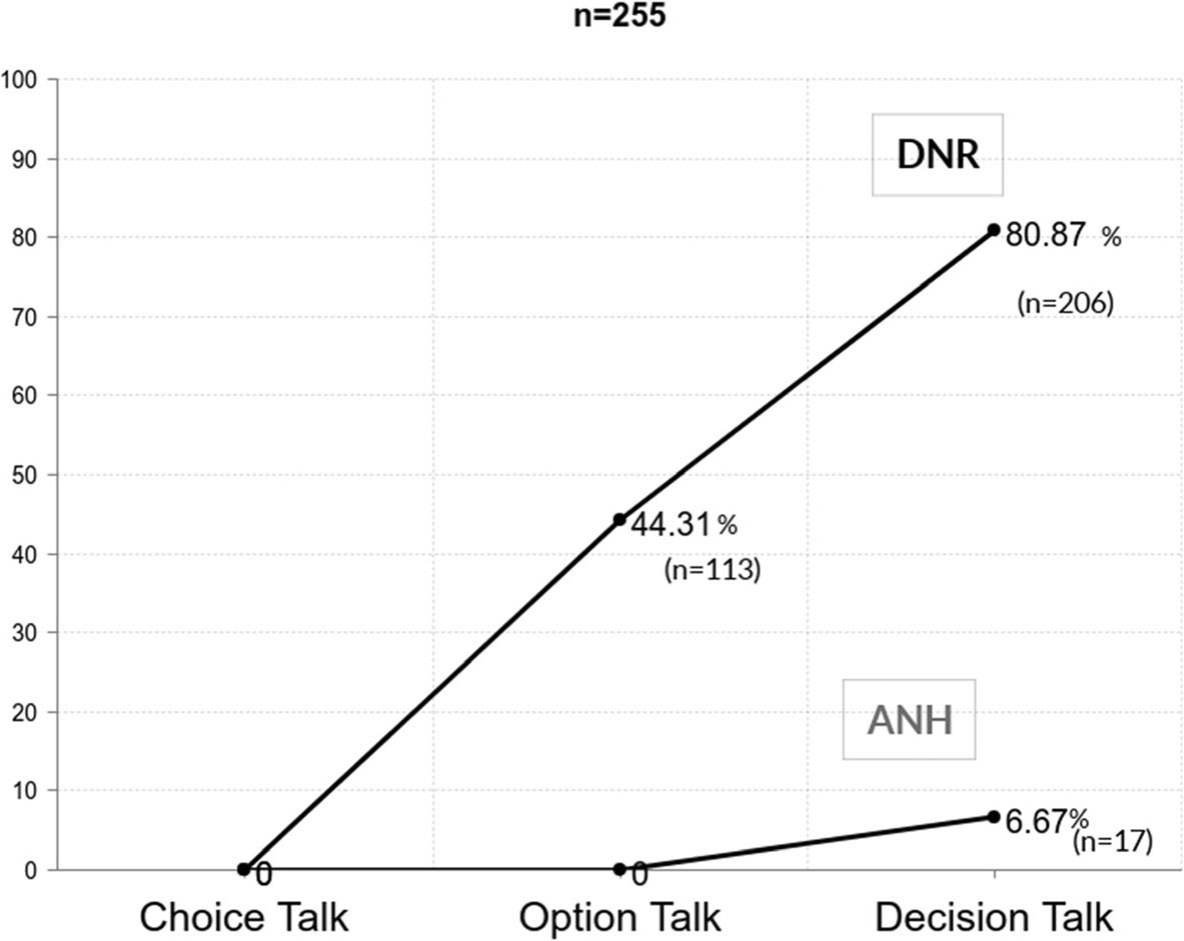
The completion rates on documentation of do not resuscitate (DNR) or artificial nutrition and hydration (ANH) in the SOP model (Shared decision making with Oncologists and Palliative care specialists)

The documentation rates of DNR and ANH preferences are demonstrated in Fig. 2. No patients had their preferences on DNR or ANH documented before joining the model. The DNR documentation rate after implementing the SOP model increased from 44.3% (*n* = 113) after the option talk to 80.9% (*n* = 206) after the decision talk. No participants documented their preferences on ANH after the option talk, and only 6.67% (*n* = 17) documented after the decision talk. The DNR documentation rate was 52.3% for patients with advanced cancer outside of the model who died in our hospital during the study period.

The grouping of participants according to the final documented status of DNR is shown in Table 1. There were significant statistical differences between the two groups on the variables of education (*p* = 0.030) and ECOG status (*p* < 0.001). In the DNR documentation group, 65 patients (31.6%) graduated from “University or above” and 56 patients (27.2%) finished senior high school. In the patients who did not have DNR documentation, 21 patients (42.9%) had the education level of “elementary school or below” and 10 patients (20.4%) finished junior high school. For ECOG performance status, 65 patients (31.6%) were ECOG 3 and 75 patients (35.4%) were ECOG 2 in the DNR documented group. Meanwhile, 29 patients (59.2%) who didn’t complete DNR documentation were ECOG 0 and 1.

Results of the univariate and multivariable logistic regression analyses to address the association between the documentation of DNR and the variables are shown in Table 2. Education (*p* = 0.015) and ECOG performance status (*p* = 0.001) were significant predicting variables in the univariate model but only ECOG performance status (*p* = 0.002) was a significant associating factor in the multivariable model. A higher ECOG performance status was associated with a higher documentation rate of DNR as revealed by the odds ratios of 2.541 (95% Confidence interval [CI]: 1.185–5.449, *p* = 0.017) and 6.695 (95% CI: 2.131–21.035, *p* = 0.001) for functional status 2 and 3, respectively, comparing to the functional status 0 and 1.

**Table 2:** Logistic univariate and multivariate analysis of the variables related to DNR completion

	Univariate				Multivariate			
	OR	95% CI		*p*	OR	95% CI		*p*
		Lower	Upper			Lower	Upper	
Age	1.003	0.983	1.024	0.759	1.007	0.979	1.036	0.641
Gender (Ref: male)				0.179				
female	0.652	0.350	1.216	0.179	0.509	0.248	1.043	0.065
Education (Ref: Elementary school or below)				0.015^*^				0.052
Junior high school	0.594	0.238	1.483	0.265	0.630	0.227	1.753	0.377
Senior high school	1.847	0.774	4.411	0.167	1.824	0.675	4.929	0.236
University or above	2.111	0.888	5.016	0.091	2.604	0.903	7.508	0.077
ECOG (Ref: 0 and 1)				0.001^*^				0.002^*^
2	1.913	0.965	3.792	0.063	2.541	1.185	5.449	0.017^*^
3	7.140	2.377	21.451	0.001^*^	6.695	2.131	21.035	0.001^*^
Religion (Ref: Not specified)				0.351				0.946
Buddhism	1.017	0.426	2.427	0.970	1.052	0.402	2.751	0.917
Christianity	1.438	0.363	5.700	0.605	0.770	0.173	3.432	0.732
Taoism/traditional religions	0.738	0.322	1.689	0.472	0.852	0.344	2.112	0.730
Other	0.216	0.038	1.214	0.082	0.418	0.028	6.225	0.527

Abbreviations: *DNR* do not resuscitate; *ECOG* Eastern Cooperative Oncology Group performance status; *CI* Confidence interval; *OR* Odds ratio

^*^p < 0.05

## Discussion

The SOP model significantly increased the rate of DNR documentation in patients with advanced cancer in this pilot study. The functional status of patients is the most important influencing factor on the documentation rate in this model. The coordination of oncologists and palliative care specialists in the SOP model demonstrated a feasible strategy to facilitate the decision-making process of the patients. The model might help patients with advanced cancer to receive EOL treatments concordant with their wishes.

The SOP model increased the DNR documentation rate of participants from zero before the model to more than 80% after joining the model. At the same time, the patients with advanced cancer without joining the model only had 52.3% of documentation rate before dying in our hospital from the administrative data. The incorporation of pamphlets and the semi-structured discussion helped resolve the individual divergence on the preference of verbal vs written communication, and the model gave patients enough time for deliberation on their preferences to make decisions. Furthermore, palliative care specialists not only provide the essential components during the decision talk including understanding the patients’ informed preferences but also provide emotional support for the patients and their families when documenting the EOL care preferences. The model revealed the benefits of facilitating the documentation of EOL care preferences by the integration of palliative care specialists into the cancer treatment team for patients with advanced cancer. This type of model could be adopted in other cancer care hospitals around the world.

The first Patient Right to Autonomy Act in Asia is just enacted in Taiwan in 2019 to provide the legal basis for ACP that patients now have the right to refuse unwanted medical treatments and receive palliative care in certain clinical conditions. The Act is in line with the worldwide trend of person-centered medical care. However, despite patients with advanced cancer having a higher need for timely ACP especially in the communication of preferences of DNR and ANH than the general population, the study revealed a low documentation rate of the preferences in these patients before joining the model. Clearly, it is important for the government and the medical professionals to promote ACP especially in patients with advanced cancer. The results that more than 80% of patients who joined the model expressed their EOL care preferences in the terminal stage demonstrated that the model is effective in the documentation of these preferences. The dissemination and promotion of the SOP model which incorporates both oncologists and palliative care specialists are warranted in countries with laws on ACP.

The low documentation rate of ANH preferences demonstrated the complexity of this issue. ANH was identified as a prevailing ethical dilemma in our previous surveys of health professionals on their attitude toward EOL care [37, 38]. The supplementation of ANH may relieve discomfort caused by dehydration, but the requirements of tubes or catheters may increase the burden for patients [39–41]. Whether ANH is beneficial as a basic nutritional supplement or may become problematic due to the possibility of fluid overload in patients with advanced-stage cancer remains an area of divergent opinions among clinicians [42, 43]. As a result, it is difficult for patients and families to form decisive preferences on ANH even after the present SOP model. Further cooperation of a multi-disciplinary team with oncologists, palliative care specialists, physiatrists, nurses, and nutritionists is essential to develop ANH management guidelines. After establishing practice guidelines, it is important to not only incorporate the evidence to the design of decision aids but to also address cultural and religious concerns to promote a consensus between appropriate treatment choices and the patients’ preferences.

A previous study on cancer patients demonstrated that age, gender, education, and functional status were associated with the willingness to participate in ACP [44]. Our study showed that functional status was the statistically significant factor associated with the documentation rate on DNR preference. A higher ECOG means a more deteriorated physical function, and it was correlated with a higher documentation rate when facing the EOL. Patients may associate functional decline with the threat of death, and the stress may prompt the patients to address their care preferences at the EOL [30]. The change of functional status may be a suitable point in the continuum of cancer care to implement ACP in oncology practice [19].

The study has several limitations. First, the nonrandomized design and potential selection bias may exist so that the participants were willing to join the model because they were more aware of the importance of ACP than the general public. Nonetheless, the present study served the purpose of model testing. Second, given that many participants were still alive during the period of the analysis, whether the increased documentation rate on DNR is associated with the higher receipt of concordant care at the EOL needs further follow-up study. Third, the study is conducted in a region where Confucian culture predominates and some results may require modifications when applying to other countries.

## Conclusions

The SOP model with the integration of oncologists and palliative care team significantly increased the documentation rate of DNR preferences in patients with advanced cancer in this pilot study. The process of shared decision making might help patients receive EOL care according to their wishes. The model dissemination will be useful for the countries having the laws on ACP such as the Patient Right to Autonomy Act in Taiwan. The model may be applied in the cancer treatment course in the era of person-centered care, and it could be adopted in cancer care hospitals worldwide.

## Supplementary information

**Additional file 1.** The interview guide of “option talk”.

## Data Availability

The datasets analyzed in the present study are available from the corresponding author on reasonable request.

## Abbreviations

ACP: Advance care planning
ANH: Artificial nutrition and hydration
DNR: Do not resuscitate
ECOG: Eastern Cooperative Oncology Group
EOL: End of life
GRADE: Grading of Recommendations Assessment, Development and Evaluation
SDM: Shared decision making
SHARE model: Seek participation, Help comparison, Assess values, Reach decision, Evaluate decision
SOP model: Shared decision making with Oncologists and Palliative care specialists
