# Supplementary material for: Shared decision making with oncologists and palliative care specialists effectively increases the documentation of the preferences for do not resuscitate and artificial nutrition and hydration in patients with advanced cancer: a model testing study
Source: BMC Palliat Care. 2020 Feb 4;19:17. doi: 10.1186/s12904-020-0521-7 (PMC7001377; doi:10.1186/s12904-020-0521-7)
Supplement: Supplementary file 1 — Additional file 1. The interview guide of “option talk”. [file 12904_2020_521_MOESM1_ESM.docx]

Interview guide of the “option talk”

Three themes

1. rapport building and communication between patients and physicians

“How is the experience of cancer treatment in the hospital?”

1. symptoms and adverse effects due to current anti-cancer treatment

“Did you have any adverse discomforts in past days during the treatment course?”

(Follow up questions: Did you inform your care teams? How did the discomforts be managed?)

1. EOL care preferences.

“Have you considered the care preferences toward the EOL such as whether you would choose to be cardio-pulmonary resuscitated?”

“Have you considered to receive clinically assisted hydration via nasogastric tube, gastrostomy, or intravenous administration toward the EOL?”.
